# Concussion Knowledge Among Neurosurgery, Neurology, and Emergency Medicine Residents: A Multi-institutional Study in the Western Region of Saudi Arabia

**DOI:** 10.7759/cureus.80426

**Published:** 2025-03-11

**Authors:** Abdulaziz M Alghamdi, Mahmoud A Fallatah, Abdullah AlMansour, Abdulaziz N Aljohani, Alaa Ashqar, Ahmed I Lary

**Affiliations:** 1 College of Medicine, King Saud Bin Abdulaziz University for Health Sciences, Jeddah, SAU; 2 Research, King Abdullah International Medical Research Center, Jeddah, SAU; 3 Department of Neurosurgery, Prince Sultan Military Medical City, Riyadh, SAU; 4 Department of Orthopaedic Surgery, Ministry of the National Guard-Health Affairs, Jeddah, SAU; 5 Department of Emergency Medicine, King Abdulaziz University Hospital, Jeddah, SAU; 6 Department of Neurosurgery, Ministry of the National Guard-Health Affairs, Jeddah, SAU

**Keywords:** concussion, emergency medicine, neurology, neurosurgery, resident

## Abstract

Background: Despite the major prevalence of concussion, it is the most misdiagnosed and undertreated form of traumatic brain injury.

Methods: This multi-institutional questionnaire-based cross-sectional study aimed to assess concussion knowledge, exposure, and learning among neurosurgery, neurology, and emergency medicine residents in the western region of Saudi Arabia. The data collection of the responses was conducted between January and March 2024. The questionnaire contained 30 structured questions in three sections: Demographic data, knowledge of concussion definitions and management, and learning experiences on the topic.

Results: A total of 105 residents participated, with a mean age of 28.32±2.62 years. Fifty-two (49.52%) were males. Neurosurgery residents scored significantly higher, 4±0.85 out of 9, in concussion knowledge in comparison to residents in neurology, 3 ± 1.32 out of 9, and emergency medicine, 3.32±1.06 out of 9 residents. These differences were statistically significant (p=<0.005). Linear regression analysis indicated that residents who received lower scores on the concussion knowledge tended to rate themselves lower than those who received higher scores (B=0.461, p=0.0107). Fifty-six (53.33%) residents have not been clinically exposed to patients with concussions. The residents scored a median of 8 (2-10) out of 10 regarding their desire to involve concussion-related knowledge in their curricula. Fifty-seven (54.29%) residents chose textbooks as their most preferred source of learning about concussion, and 37 (35.24%) chose textbooks as their most preferred format.

Conclusion: Residents of three specialties exhibited notable gaps in their knowledge of concussion; however, neurosurgery residents demonstrated better knowledge than their counterparts. These findings necessitate further education and training according to residents’ preferred sources and formats to improve medical care and reduce unfavorable outcomes.

## Introduction

There is considerable ambiguity when mentioning the term concussion to patients, care providers, and even some physicians. This definition can be vague because of the lack of a globally accepted single definition of concussion [1]. However, this is changing owing to the extensive amount of research and data gathered in recent years [2]. A traumatic brain injury (TBI) is a disruption in the normal function of the brain that can be caused by a blow, a jolt to the head, or even a penetrating head injury. It typically includes temporary impairment of neurological function, which heals spontaneously over time. Many physicians consider concussion a transient and immediate symptom of a TBI [3,4]. However, many others struggle to translate this definition into medical practice because of misunderstandings [4]. Therefore, some claim that this vague definition, while widely accepted, is too broad and does not address important variables, such as the mechanism, severity score, or duration of symptoms.

Another definition by the Quality Standards Subcommittee of the American Academy of Neurology (AAN) defines concussion as a trauma-induced change in mental status that may or may not include loss of consciousness; in more than two-thirds of diagnosed concussions, there is no loss of consciousness (LOC) [3,5]. Furthermore, several organizations have offered more detailed definitions; however, none of them are universally accepted. According to the World Health Organization, it is estimated that 6 out of 1,000 people are affected by concussions annually [4]. Locally, underreporting in Saudi Arabia is widely noted, and it is a major obstacle to estimating its incidence and conducting meaningful research [6]. 

The likelihood of a physician managing a concussion case is high, considering that it is a common injury, especially in pediatric patients [7]. Unfortunately, the lack of Class I evidence to guide the management makes the physician depend on his or her knowledge of current guidelines and experience [8]. This potentiates the chance of suboptimal outcomes. The diagnosis of concussion is primarily based on symptomatology when one or more symptoms are required to make a diagnosis [9]. The clinical findings in patients with a concussion include dizziness, headache, loss of concentration, nausea, vomiting, disorientation, blurry vision, difficulty speaking, impaired memory, loss of balance and coordination, emotional liability, tingling sensation, LOC, or amnesia [10-14]. Symptoms and signs vary from case to case and can be easily overlooked. It is strongly recommended currently for all concussed individuals to seek professional medical care [15]. Although most individuals who have a concussion have an excellent prognosis, in some patients, the recovery from a concussive injury is not followed by a typical progressive improvement [16]. 

Various factors can elevate the risk of prolonged morbidity following a concussion, including repeated concussions that occur with decreasing impact force, progressively slower recovery after each concussion, multiple concussions within a short period, and concussions that result in an extended or incomplete recovery. All of these factors should raise the concern of clinicians about the possible development of catastrophic or long-term sequelae [17]. One of the acute catastrophic sequelae of concussion is the second impact syndrome [18,19]. In addition, serious long-term sequelae include postconcussion syndrome and chronic traumatic encephalopathy [20,21]. Several studies in the literature have assessed and reported concussion knowledge among medical residents, medical students, coaches, and parents. Many of those studies showed knowledge gaps among the participants, all of which were international studies [6,9,22,23]. To the best of our knowledge, there are no local studies assessing the level of knowledge of concussions among clinicians or the general population in Saudi Arabia.

In summary, concussions are the most likely type of TBI that can be missed or incorrectly diagnosed [24]. Doctors must have adequate knowledge to assess and diagnose concussions, mitigate injury-related complications, and prevent long-term consequences. This includes recognizing signs of concussive brain injuries, monitoring symptoms, and ensuring patients avoid secondary head trauma and premature return to activity before full recovery [9]. This study aimed to assess the knowledge of concussion definition, diagnosis, management, and consequences among neurosurgery, neurology, and emergency medicine residents at all levels of programs in the western region of Saudi Arabia to identify the knowledge gaps that may exist.

## Materials and methods

This cross-sectional study was conducted in the western region of Saudi Arabia in January- March 2024. This study aimed to evaluate the knowledge of concussion syndromes among neurosurgery, neurology, and emergency medicine residents at all levels of programs in different cities in the western region of Saudi Arabia. Residents rotating from other residency programs, withdrawn residents, and board-certified physicians were excluded from this research. Informed consent was obtained from all participants before the study. The participants were given a predesigned, self-administered, and validated questionnaire adapted from a similar study conducted in Canada by Boggild and Tator [9]. The questionnaire was modified slightly to accommodate the research objectives. The modifications did not exceed a 10% change from the original survey, none of which was related to the core questions on knowledge about concussions. We conducted a pilot study with 25 residents to assess the reliability and validity of the questionnaire. None of the residents involved in the pilot tests were included in this study. The modified version has been attached to the supplementary data (Appendix A).

The questionnaire contained 30 structured questions in three sections: demographic data, knowledge of concussion definitions and management, and learning experiences on the topic. The demographic data section contained 12 questions about gender, residency program, place of medical school, participation in sports and recreation, and personal history of concussions. The knowledge of concussion definitions and management section contained nine questions assessing participants’ knowledge of concussion definitions and management, based on the 2008 consensus statement on concussion in the 3rd international conference on concussion in sport held in Zurich. In the second section, respondents were given a score from 0 to 9 based on their answers to 9 knowledge-based questions (Questions 13 to 21). Each question was marked as correct or incorrect, with one point indicating the correct answer. No partial marks were awarded. If a question required the selection of multiple correct answers, all correct options had to be selected, and incorrect options did not receive any points.

Finally, in the third section, nine questions in the learning experiences section gathered information regarding clinical exposure to concussions, previous learning about concussions, preferred format and source for physician learning, self-ranking of knowledge about concussions, motivation to learn more about the topic, and any challenges that might be faced when diagnosing and managing patients with concussions.

The questionnaire was distributed online using Google Forms in January 2024. No names were taken from the participants. The study was approved by the Institutional Research Board of KAIRMC, KSAU-HS, Riyadh (IRB/2270/22). The targeted population consisted of 301 residents, distributed as follows: 48 neurosurgery residents, 98 neurology residents, and 155 emergency medicine residents. The required sample size was calculated to be 170 participants, with a 95% confidence level and a margin of error of ± 5%, assuming a response distribution of 50%. The final sample size was 187 subjects, accounting for a 10% nonresponse rate. The Raosoft software website was used for the calculations. For statistical analysis, chi-square or Fisher’s exact test was used for categorical data, whereas t-tests or ANOVA were used for numerical data. Linear regression was used to describe the relationship between self-ranked concussion knowledge scores and actual concussion knowledge scores based on the assessment of Questions 13 to 21. Statistical significance was set at p<0.05. The data were analyzed using JMP software version 15.0 on John’s Macintosh Project. 

## Results

Of the total number of 301 residents, 105 (34.9%) agreed to participate in this study and completed the survey, accounting for a 56.14% response rate out of the initially calculated sample size of 187. Of the total specialties, 23 (47.9%) out of 48 neurosurgery residents, 33 (33.67%) out of 98 neurology residents, and 49 (31.6%) out of 155 emergency medicine residents participated in this study. Among all residents, the mean age was 28.32±2.6 years, and the total number of male residents was 52 (49.5%) (Table 1). Almost one-third of the residents, with a total of 31 (29.5%), were in their postgraduate year (PGY) one, followed by 24 (22.85%) residents in PGY 3 and 20 (19.0%) residents in PGY 4. PGY 5 had only 10 (9.5%) neurosurgery and neurology residents, and PGY 6 had only 4 (3.8%) neurosurgery residents. Almost all the residents, 102 (97.1%), had participated in sports in the past 2 years. The median of sports recreation times in the last week was one (1-6) time, and one-third of the residents, 35 (33.3%), spent 31-60 minutes on each occasion. The rest of the demographic and sports recreation details for each specialty are shown in Table 1. 

**Table 1:** Demographics and sports recreation of neurosurgery, neurology, and emergency medicine residents. ^*^number, ^†^standard deviation, ^‡^postgraduate year

Variables	All residents, n=105	Neurosurgery residents, n=23	Neurology residents, n=33	Emergency medicine residents, n=49
Male, n^*^ (%)	52 (49.5%)	15 (65.2%)	14 (42.4%)	23 (46.9%)
Age (in years), mean ± SD^†^	28.32±2.62	29±1.59	27.75±3.03	28.38±2.69
Levels of training, n^*^(%)				
PGY^‡^ 1	31 (29.5%)	2 (8.7%)	15 (45.45%)	14 (28.6%)
PGY^‡^ 2	16 (15.2%)	5 (21.7%)	3 (9.1%)	8 (16.3%)
PGY^‡^ 3	24 (22.85%)	4 (17.4%)	7 (21.2%)	13 (26.5%)
PGY^‡^ 4	20 (19.0%)	2 (8.7%)	4 (12.1%)	14 (28.6%)
PGY^‡ ^5	10 (9.5%)	6 (26.1%)	4 (12.1%)	-
PGY^‡^ 6	4 (3.8%)	4 (17.4%)	-	-
Sports recreation in the last 2 years, n^*^ (%)	102 (97.1%)	23 (100.0%)	30 (90.9%)	49 (100.0%)
Sports recreation times in the last week, median (min-max)	1 (1-6) times	2 (1-6) times	1 (1-4) times	1 (1-6) times
Time spent on each occasion, n^*^ (%)				
1 to 15 minutes	33 (31.4%)	6 (26.1%)	13 (39.4%)	14 (28.6%)
16 to 30 minutes	22 (20.95%)	5 (21.7%)	8 (24.2%)	9 (18.4%)
31 to 60 minutes	35 (33.3%)	9 (39.1%)	7 (21.2%)	19 (38.8%)
More than one hour	15 (14.3%)	3 (13.0%)	5 (15.15%)	7 (14.3%)

**Figure 1:**
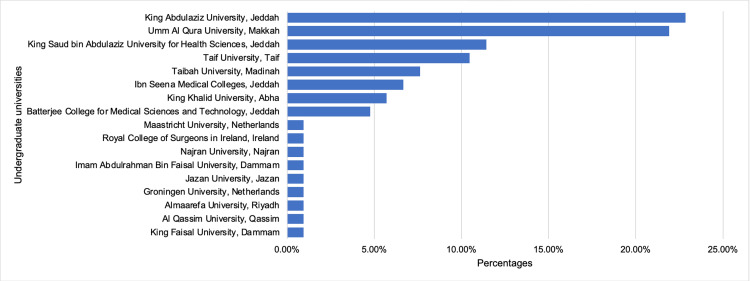
Bar chart of undergraduate universities' percentages of the residents.

Twenty-four residents (22.9%) had undergraduate degrees from King Abdulaziz University, Jeddah, closely followed by 23 residents (21.9%) from Umm Al Qura University, Makkah (Figure 1).

The residents scored a mean of 3.37±1.2 out of 9 in concussion knowledge. However, based on specialty, neurosurgery residents had a significantly higher mean score of 4±0.85 than neurology and emergency medicine residents, who had mean scores of 3±1.3 and 3.32±1.0, respectively (p*=*0.005) (Table 2). 

**Table 2:** Knowledge of concussion syndrome among neurosurgery, neurology, and emergency medicine residents. Questions are mentioned in the appendix table. ^*^number, ^†^standard deviation, ^‡^ the statistical test ANOVA was used to obtain the f-value (p-value of<0.05 was considered statistically significant).

Variables	All residents, n=105	Neurosurgery residents, n=23	Neurology residents, n=33	Emergency medicine residents, n=49	p-value^ ‡^
Question 13, the definition of concussion, n^*^(%)	70 (66.7%)	20 (86.95%)	19 (57.6%)	31 (63.3%)	-
Question 14, definition of concussion, n^*^(%)	86 (81.9%)	23 (100.0%)	24 (72.7%)	39 (79.6%)	-
Question 15, definition of concussion, n^*^(%)	55 (52.4%)	13 (56.2%)	14 (42.4%)	28 (57.1%)	-
Question 16, symptoms of concussion, n^*^(%)	7 (6.7%)	1 (4.3%)	5 (15.15%)	1 (2.0%)	-
Question 17, symptoms of concussion, n^*^(%)	60 (57.1%)	13 (56.2%)	19 (57.6%)	28 (57.1%)	-
Question 18, mechanism of concussion, n^*^(%)	56 (53.3%)	15 (65.2%)	14 (42.4%)	27 (55.1%)	-
Question 19, management of concussion, n^*^(%)	7 (6.7%)	3 (13.0%)	1 (3.0%)	3 (6.1%)	-
Question 20, concussion red flags, n^*^ (%)	7 (6.7%)	3 (13.0%)	2 (6.1%)	2 (4.1%)	-
Question 21, consequences of repetitive concussive injury, n^*^ (%)	6 (5.7%)	1 (4.3%)	1 (3.0%)	4 (8.2%)	-
Total, mean±SD^†^	3.37±1.16	4±0.85	3±1.32	3.32±1.06	<0.005
Self-ranked concussion knowledge, mean±SD^†^	4.8±2.15	7.08±1.37	3.7±1.62	4.44±2.01	<0.001

Regarding the personal history of concussions, 20 (19.0%) residents reported having one or more concussions during their lifetimes, and 11 (55.0%) reported sports or recreational activity as the causative factor for at least one concussion. In addition, 23 (21.9%) residents reported having a person close to them having one or more concussions, and 14 (60.9%) also reported sports or recreational activity as the causative factor for at least one concussion. Regarding clinical exposure, 21 (91.3%) neurosurgery residents reported having seen a patient with a concussion in the acute phase, which was significantly higher than 7 (21.2%) and 29 (59.2%) reported by neurology residents and emergency medicine residents, respectively (p=<0.001). Moreover, 17 (73.9%) neurosurgery residents reported having seen a patient with postconcussive syndrome, which was significantly higher than that of neurology residents and emergency medicine residents, who reported 8 (24.2%) and 13 (26.5%), respectively (p<0.003). When asked how many cases of concussions they usually encounter per month, 56 (53.3%) residents reported that they did not encounter any cases, and neurosurgery residents were the only ones who reported encountering 6-10 cases and more than 10 cases of concussions per month (Table 3). Gender, positive personal history of concussion, and number of concussion cases encountered per month were not associated with the total concussion knowledge scores of neurosurgery, neurology, and emergency medicine residents. However, there was a significant association between a positive history of concussion in a close person and higher concussion knowledge scores in neurosurgery residents only (p=0.017), as neurology and emergency medicine residents showed no significant associations. 

**Table 3:** Concussion history and clinical exposure of neurosurgery, neurology, and emergency medicine residents. ^*^numbers, ^†^the statistical test ANOVA was used to obtain the f-value (a *P* value of < 0.05 was considered statistically significant)

Variables	All residents, n = 105	Neurosurgery residents, n = 23	Neurology residents, n = 33	Emergency medicine residents, n = 49	p- value^†^
Personal history of one or more concussions, n^*^(%)	20 (19.0%)	4 (17.4%)	7 (21.2%)	9 (18.4%)	
History of one or more concussions in a close person, n^*^ (%)	23 (21.9%)	8 (34.8%)	5 (15.15%)	10 (20.4%)	
Having seen a patient with concussion in the acute phase, n^*^(%)	57 (54.3%)	21 (91.3%)	7 (21.2%)	29 (59.2%)	<0.001
Having seen a patient with post-concussive syndrome, n^*^ (%)	38 (36.2%)	17 (73.9%)	8 (24.2%)	13 (26.5%)	<0.003
how many cases of concussion per month do you encounter? n^*^ (%)					
None	56 (53.3%)	4 (17.4%)	28 (84.8%)	24 (49.0%)	-
1-5 cases	41 (39.0%)	11 (47.8%)	5 (15.15%)	25 (51.0%)	-
6-10 cases	3 (2.85%)	3 (13.0%)	0 (0%)	0 (0%)	-
More than 10 cases	5 (4.8%)	5 (21.7%)	0 (0%)	0 (0%)	-

**Figure 2:**
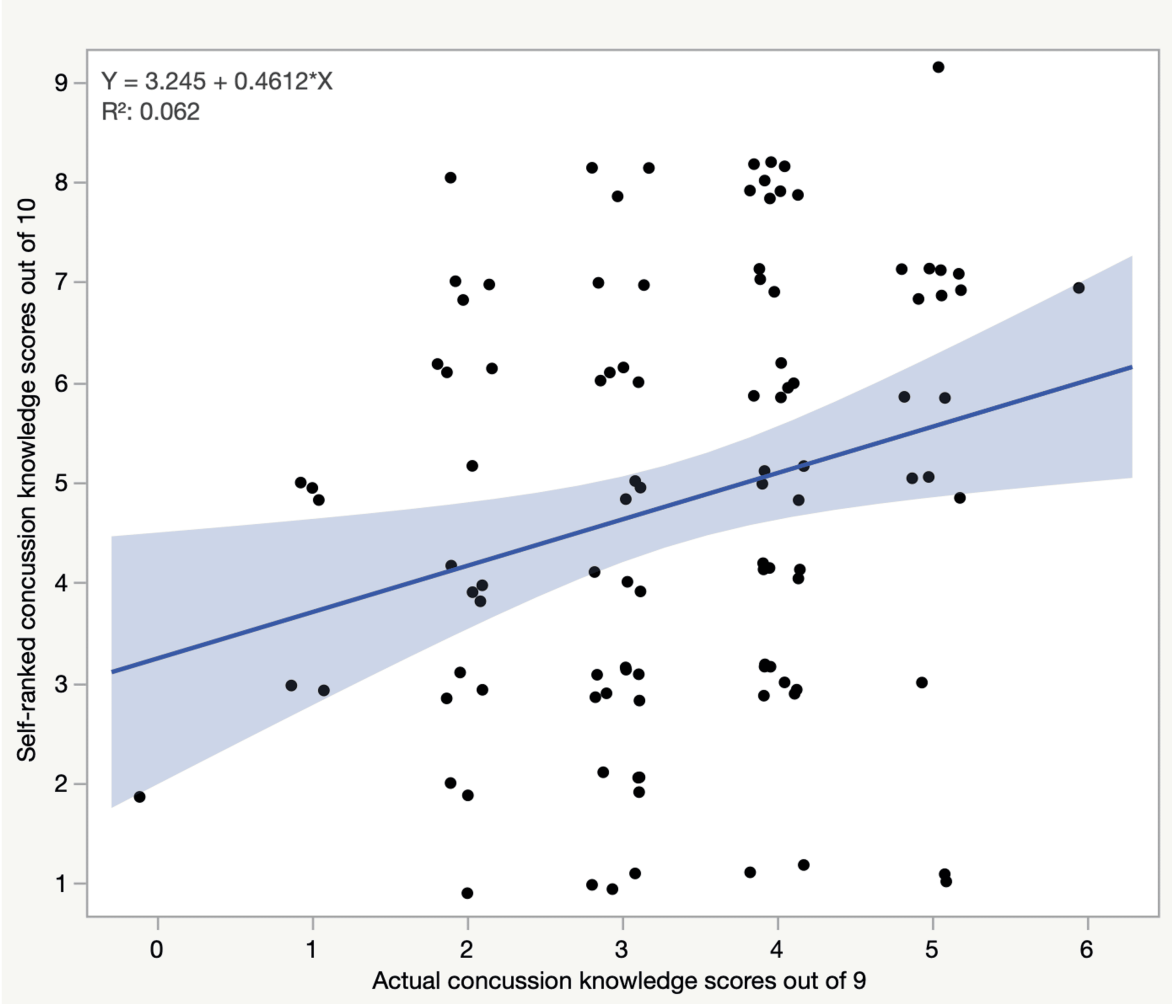
The relation between actual scores and self-ranked scores of the residents.

Regarding self-ranked concussion knowledge, the neurosurgery residents tended to rank themselves significantly higher, with a mean self-ranked score of 7.08±1.4, than the neurology and emergency medicine residents, who had mean self-ranked scores of 3.72±1.6 and 4.44±2.0, respectively (p<0.001) (Table 2). However, the linear regression analysis showed that residents who scored poorly tended to self-rank themselves lower than those who scored higher on the self-ranked concussion knowledge scores, which significantly predicted the actual concussion knowledge scores (B=0.461, p=0.0107) (Figure 2). 

**Figure 3:**
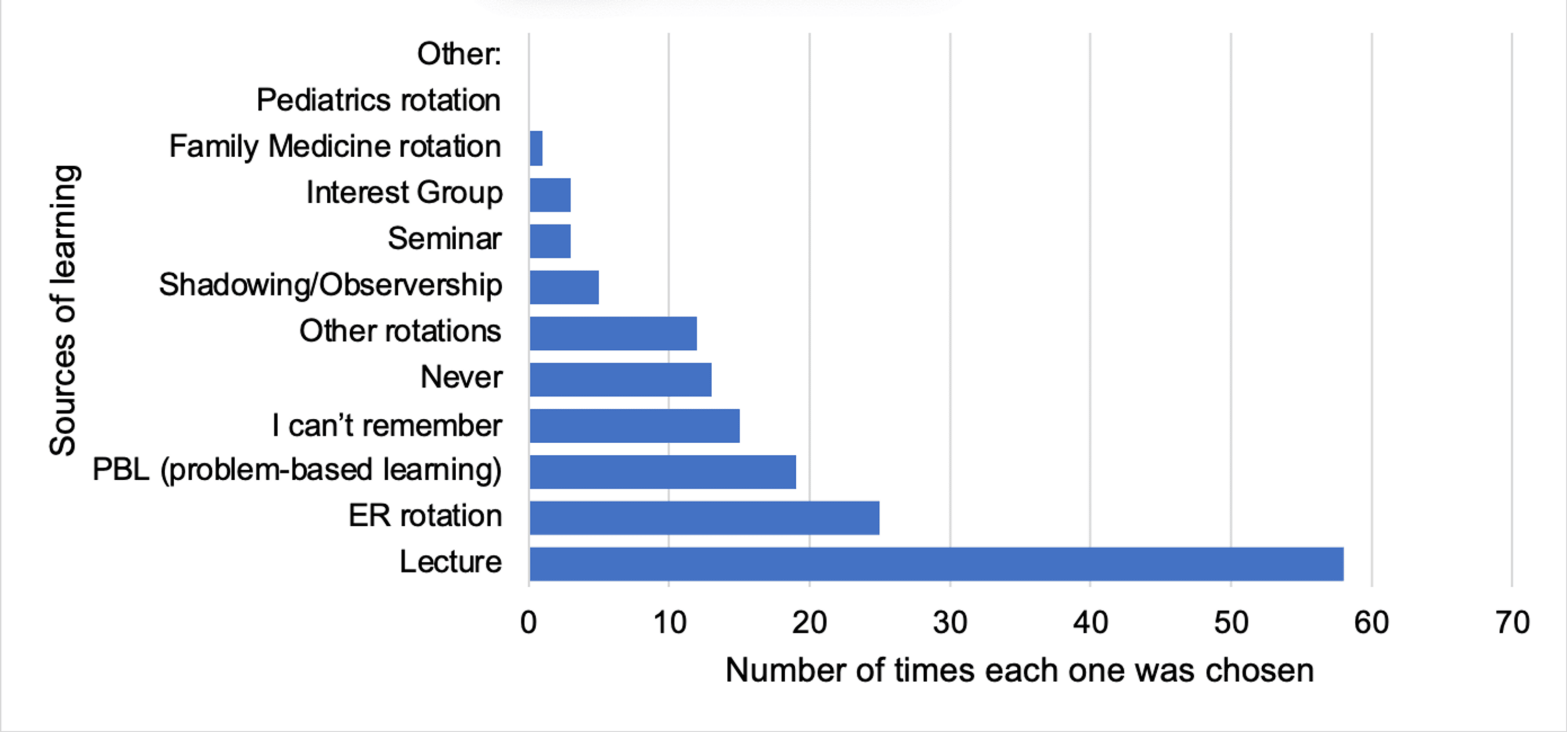
Bar chart of sources of learning about concussion in undergraduate years (participants were allowed to choose more than one answer).

When asked about the sources of learning about concussions in their undergraduate years, 58 (37.7%) of the residents chose lecture, 25 (16.23%) chose emergency medicine rotation, and 19 (12.34%) chose problem-based learning (Figure 3). 

Table 4 shows the seven most relevant written answers by the residents regarding the challenges of diagnosing and managing concussions. 

**Table 4:** Challenges of diagnosing and managing a concussion among the residents (an open-ended question).

Answers
The patient believes, especially when the symptoms, such as headache, stayed for a long period of time, so you need extra effort to explain to them the pathophysiology of a concussion, the expectations about the recovery period, and the risk of second impact syndrome.
Convincing the patient and their family of the following: the symptoms are expected to last for a few days or longer, there’s only symptomatic management until the patient’s symptoms resolve, there’s no need for a follow-up CT brain unless there’s worsening of symptoms, and that the patient doesn’t need to be admitted to the hospital for a concussion.
Busy emergency departments with a lack of available beds to be reserved for concessive patient observation.
Some patients will only be convinced that they have a brain injury when they see imaging abnormalities, which is not possible in concussions.
The decision to discharge the patient, as they can be stable at the moment and then collapse after being discharged.
The wide range of symptoms and history taking.
It is a clinical diagnosis by exclusion of any other possible pathologies.

**Figure 4:**
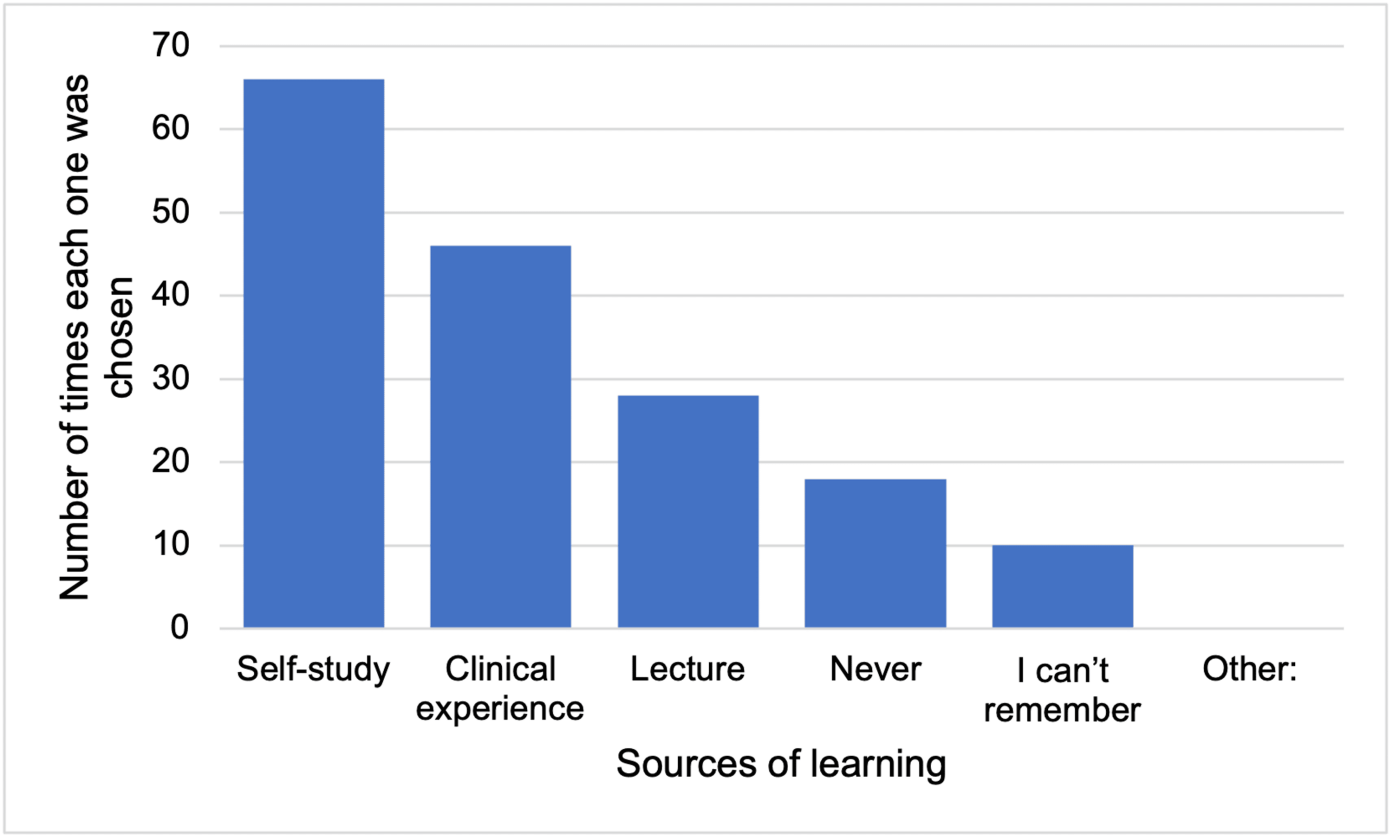
Bar chart of sources of learning about concussion in residency (participants were allowed to choose more than one answer).

When asked about the sources of learning about concussions in residency, 66 (39.5%) of the residents answered self-study, 46 (27.3%) answered clinical experience, and 28 (16.6%) answered lecture (Figure 4).

**Figure 5:**
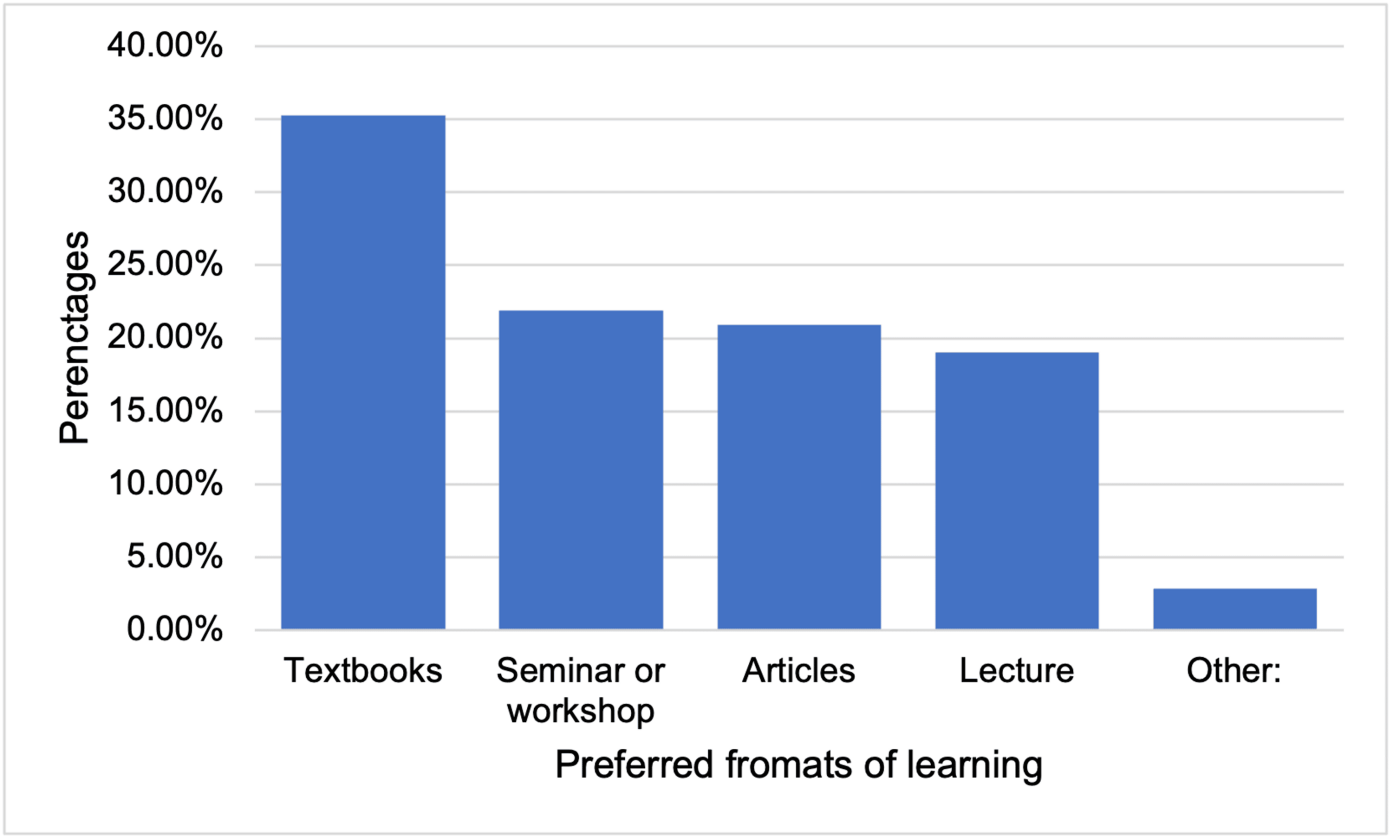
Bar chart of the preferred format for physician learning material among the residents.

The residents expressed their level of wanting to learn more about concussions in their medical curriculum in residency by having a median score of 8 (2-10) out of 10, with no significant difference between the 3 groups. The most preferred format of learning by the residents was textbooks 37 (35.2%), followed by seminars or workshop 23 (21.9%), and articles 22 (20.95%) (Figure 5).

**Figure 6:**
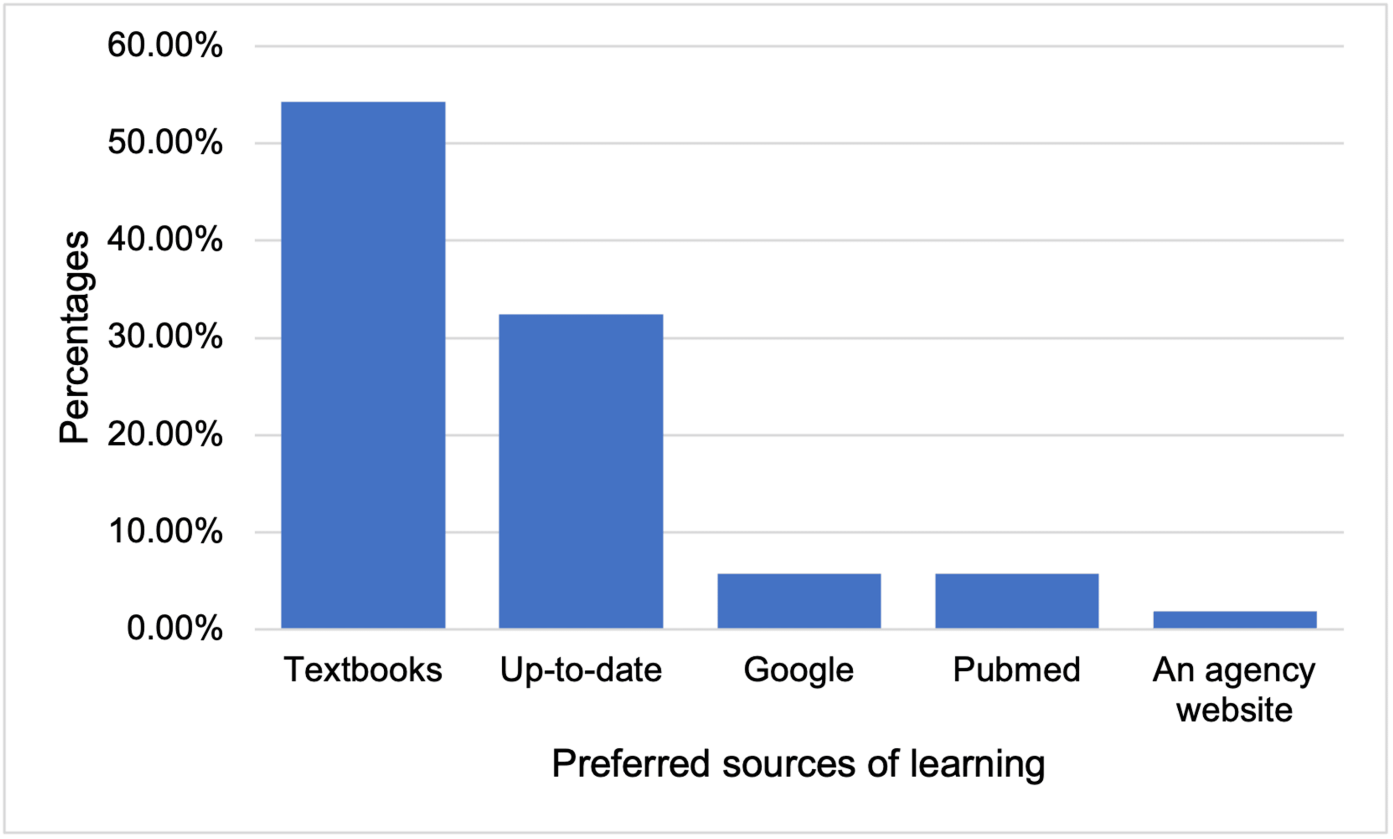
Bar chart of preferred sources of learning about concussion among the residents.

In addition, 3 (2.9%) residents who chose other mentioned infographics and short videos. The most preferred source of learning was textbooks 57 (54.3%), followed by up-to-date 34 (32.4%) (Figure 6). 

## Discussion

This study aimed to evaluate the definition, diagnosis, management, and consequences of concussion among residents of neurosurgery, neurology, and emergency medicine in the western region of Saudi Arabia. Neurosurgery residents are involved in the diagnosis and management of brain and nervous system disorders, including traumatic brain injuries such as concussions. Neurosurgeons specialize in the surgical treatment of brain and nervous system disorders. This may involve the management of more severe concussion cases that require surgical intervention. In contrast, neurology residents specialize in the medical management of brain and nervous system disorders and may manage mild-to-moderate concussion cases without surgical intervention. Additionally, emergency medicine residents are often the first to evaluate and manage patients with concussions in the emergency department. They play a critical role in the initial assessment and stabilization of patients with head injuries. This includes ruling out other more severe head injuries that may require urgent intervention. Therefore, including these three specialties in this study allowed for a comprehensive evaluation of concussion knowledge. 

This study found that neurosurgery residents had a significantly higher mean score for concussion knowledge than neurology and emergency medicine residents. However, there was a notable lack of concussion knowledge among these 3 specialties (Table 2). There could be several reasons for the low scores among the residents. One possible reason is that concussion management can be relatively vague, and many medical curricula may not have covered this topic in depth. Additionally, concussion symptoms are often subjective, and there are no objective diagnostic tests, which may make diagnosis and management more challenging. Finally, there may be a lack of clinical exposure and training opportunities for managing concussions, particularly in less severe cases [8,12]. 

The higher scores among neurosurgery residents compared to other residents might be because neurosurgery residents receive specialized training in traumatic brain injuries and surgical management of brain and nervous system disorders, which makes them more familiar with the anatomy and physiology of the brain and nervous system. This specialized training may have provided them with an advantage in understanding concussion pathophysiology and its management. Moreover, neurosurgery residents in our study were more exposed to patients with concussions and post-concussive syndrome and managed more cases of concussion per month during their training compared to neurology and emergency medicine residents, which may have also contributed to their higher scores (Table 3). 

There is a lack of local studies that have assessed concussion knowledge among medical personnel in general. However, some international studies have suggested that there is a lack of knowledge among medical students and residents regarding concussions, such as one study that was conducted on 114 medical students and residents of neurosurgery and neurology, which found a significant number of them to have incomplete knowledge about concussion diagnosis and management [9]. Moreover, another study showed that 73 family residents had substantial gaps in knowledge regarding concussion diagnosis and management [22]. Other studies have addressed the knowledge and awareness of coaches, athletes, and the general population. For example, from a total of 769 coaches training high school-aged athletes (497 high school and 272 club coaches), the club coaches were less likely to be aware of the requirements for concussion education and management plans and scored lower on specific concussion knowledge questions than high school coaches [23]. Another study of the general population assessed parents’ knowledge and awareness of sports-related concussions and validated the knowledge gaps. Although most parents had some awareness about concussions, huge misconceptions regarding the definition, presentation, and treatment remained [6]. Most of these international studies showed concussion knowledge gaps in many different settings and, therefore, are consistent with our findings. 

Among all the residents, there were major shortfalls in concussion diagnosis, management, and consequence prevention. Regarding the shortfalls in concussion diagnosis, 42.86% of the residents failed to recognize that only one symptom was sufficient to diagnose a concussion, 47.61% of the residents did not know that less than one-third of all concussions involved LOC, and 40.00% of the residents thought that direct physical contact with the head was necessary to sustain a concussion. In addition, regarding the shortfalls in concussion diagnosis, 51.42% of the residents thought that not every concussion case should be seen by a physician, and 47.61% of the residents thought that it was mandatory to perform a brain CT for each concussion case. Finally, regarding the shortfalls in consequence prevention, 72.38% of the residents did not recognize that death or disability with a second concussion before recovery from a first concussion (second impact syndrome) was a consequence of repetitive concussive injury, and 48.57% of the residents did not recognize that chronic traumatic encephalopathy was a consequence of repetitive concussive injury. These are major shortfalls, and identifying such knowledge gaps is a crucial step toward improving the delivery of adequate medical care to patients with concussions. 

Our study showed that residents who had very low scores tended to self-rank themselves lower than those who scored higher, which proves the existence of self-awareness of knowledge defects among residents (Figure 2). Another noted fact is that some residents reported having never been taught about concussions during their years in medical school or residency programs. In addition, most residents expressed their desire to learn more about concussions as part of their curricula. All of these factors indicate a real need to increase concussion education and awareness among medical personnel in both medical schools and residency programs. 

Regarding the formats and sources of learning about concussions, the most preferred format of learning by residents was textbooks, followed by seminars or workshops, and the most preferred source of learning was textbooks, followed by Up-to-date. The reported formats and sources should be considered when implementing concussions in different curricula. The most relevant reported challenges in diagnosing and managing concussions were the subjective nature of symptoms, the lack of objective diagnostic tests, families’ incorrect information about concussions, and busy emergency departments with no available beds for observing concussive patients for longer periods in case of an unexpected relapse (Table 4). These challenges and obstacles should be studied thoroughly to find appropriate solutions to overcome them and deliver the best possible medical care for such a group of patients to maximize the prevention of serious complications. 

Our study has some limitations, one of which is its small sample size. However, due to the lack of local studies in our country, we believe that our study provides a good estimate of concussion knowledge among residents of these specialties. Another limitation of our study was that only residents from institutions in the western region of Saudi Arabia were included. This fact provides more research opportunities to study concussion knowledge among residents from other regions of Saudi Arabia with a larger sample size to provide a more conclusive picture of the different aspects of potentially overlooked existing knowledge gaps. 

## Conclusions

In conclusion, neurosurgery residents had a higher knowledge level of concussion definition, diagnosis, management, and consequences than neurology and emergency medicine residents in the western region of Saudi Arabia. However, there were notable knowledge gaps and pitfalls among the three groups. The residents showed self-awareness of these knowledge gaps and expressed their desire to learn more about concussions as part of their medical curricula.

As knowledge gaps exist and there is room for increasing concussion education, we recommend implementing concussion as part of the curricula of both medical schools and residencies. Such actions can improve access to medical care and reduce unfavorable outcomes for patients with concussions. 

## Appendices

Appendix A 

**Table 5:** Concussion survey to residents.

Part 1: Demographics and sports recreation background
1- What is your gender?
Male
Female
2- What is your age?
Open number value
3- What medical school did you go to for undergraduate medical education?
King Abdulaziz University, Jeddah
King Saud bin Abdulaziz University for Health Sciences, Jeddah
Batterjee College for Medical Sciences and Technology, Jeddah
Ibn Seena Medical Colleges, Jeddah
Umm Al Qura University, Makkah
Taif University, Taif
Taibah University, Madinah
King Saud University, Riyadh
King Fahd Medical City, Riyadh
Alfaisal University, Riyadh
King Saud bin Abdulaziz University for Health Sciences, Riyadh
Al-Imam Mohammed bin Saud Islamic University, Riyadh
King Faisal University, Dammam
King Khalid University, Abha
Al Qassim University, Qassim
King Faisal University, Al Hassa
Jazan University, Jazan
Najran University, Najran
Tabuk University, Tabuk
Al Jouf University, Al Jouf
Hail University, Hail
Other
4- What residency program and year are you in?
Neurosurgery
Neurology
Emergency medicine
5- What is your level of training in your residency program?
R1
R2
R3
R4
R5
6- Have you done any of the following in the past 2 years? Mark all that apply.
Football
Basketball
Volleyball
Boxing
Gymnastics
Swimming
Cycling
Horseback riding
Tennis
Running
Other
7- Last week, how many times did you participate in sports or physical activity?
Options for 1 time to 7 times given
8- About how much time did you spend on each occasion?
1 to 15 minutes
16 to 30 minutes
31 to 60 minutes
More than one hour
9- In the past, have you ever suffered a concussion? You may have been “knocked out”, knocked unconscious, confused, or had your “bell rung”. You may have felt lightheaded, not knowing where you were, etc.
Yes – once
Yes – 1-5 times
Yes – more than 5 times
No
10- If you answered yes to the previous question, how did your concussion(s) occur? Please select all that apply.
Work-related
Motor Vehicle Crash
Sports or recreational activity
Fall
Other
11- In the past, has anyone close to you suffered a concussion? They may have been “knocked out”, knocked unconscious, confused, or had their “bell rung”. they may have felt lightheaded, not knowing where they were, etc.
Yes - once
Yes - 1-5 times
Yes – more than 5 times
No
12- If you answered yes to the previous question, how did most of their concussion(s) occur? Please select all that apply.
Work related
Motor vehicle crash
Sport or recreational activity
Fall
Other
Part 2: Knowledge questions about concussions (Answers that were considered correct are in italics and underlined):
13- What is the definition of concussion? Select the best answer.
Loss of consciousness for <5 mins after an impact to the head
A complex pathophysiological process affecting the brain, induced by traumatic biomechanical forces
A structural brain injury caused by mild traumatic force that transiently decreases cerebral blood flow
14- Is a concussion a brain injury? Select the best answer.
No, as there is no abnormality seen on standard structural neuroimaging
No, as symptoms are only psychological in nature
Yes, as there is a functional disturbance that cannot be seen on standard neuroimaging
Yes, as there is structural abnormality seen on standard neuroimaging
15- Which one of the following is true?
A period of unconsciousness is necessary for the diagnosis of a concussion
Over 2/3 of all concussions involve loss of consciousness (LOC)
1/3 to 2/3 of all concussions involve loss of consciousness (LOC)
Less than 1/3 of all concussions involve loss of consciousness (LOC)
16- Which of the following is a sign or symptom of a concussion? Select all that apply.
Headache
Hemiparesis
Dizziness
Confusion
Fixed dilated pupil
Nausea and/or Vomiting
Vertigo
Amnesia
Tinnitus
Emotional or personality changes
Papilledema
Intention tremor
Fatigue
Temporary loss of consciousness
Prolonged coma
17- How many symptoms of a concussion are required to diagnose a concussion?
One or more symptoms
Three or more symptoms
Five or more symptoms
18- Which of the following is true regarding the mechanism of concussion?
Direct physical contact to the head is necessary to sustain a concussion
Localized damage to the brainstem is the cause a concussion
Localized damage to the prefrontal cortex is the cause of a concussion
Localized damage to the hippocampus is the cause of a concussion
A whiplash effect to the brain caused by an impact to any part of the body may cause a concussion
19- What is the appropriate management of concussion? Select all that apply. to be discussed
Every concussed individual should see a physician
A concussed player can return to play in the same game or practice if examined by a physician
A stepwise increase in exercise and activity if symptomatic
Physical rest is always recommended after a concussion
Mental rest is always recommended after a concussion
Signs and symptoms should be monitored for increasing severity
Full neurological exam at initial assessment is recommended
The standard mini mental status exam at initial assessment as an adequate cognitive test for concussion
MRI of the brain is mandatory
CT of the brain is mandatory
20- What are some “red flags” that may predict the potential for more prolonged symptoms and may influence your investigation and management of concussion? Select all that apply
Nose bleed
Prolonged loss of consciousness
Number and duration of symptoms
Repeated concussions occurring with progressively less impact force
Slower recovery after each successive concussion
Repeated concussions over time
Concussions close together in time
Being hit on the left side of the head
21- What are the long-term consequences of repetitive concussive injury? Select all that apply.
Dementia
Depression
Headaches
Increased risk of hemorrhagic stroke
Death or disability with a second concussion before recovery from a first concussion
Increased risk of schizophrenia
Prolonged fatigue
Impairment of concentration and memory
Parkinsonism
Chronic traumatic encephalopathy
Part 3: Learning needs about concussions
22- In your undergraduate medical education, how did you learn about concussions? Select all that apply.
Lecture
PBL (problem-based learning)
Seminar
Interest Group
Shadowing/Observership
ER rotation
Family Medicine rotation
Paediatrics rotation
Other rotations
Other
Never
I can’t remember
23- In your residency to date, how did you learn about concussions? Select all that apply.
Clinical experience
Self-study
Lecture
Never
I can’t remember
Other
24- To date, have you seen a patient with a concussion in the acute phase?
Yes
No
I don’t know
Post-concussive syndrome?
Yes
No
I don’t know
On average, how many cases of concussion per month do you encounter in your clinical practice?
None
1-5 cases
6-10 cases
More than 10 cases
25- How would you self-rank your knowledge about concussions?
Inadequate to completely adequate 1-10
26- What resource would you most likely use to find information about concussions?
Google
Up-to-date
Pubmed
Textbooks
An agency website
Other
27- Are concussions something you want to learn more about as part of your medical curriculum?
Not at all to very much 1-10
28- What is your preferred format for physician learning material?
Textbooks
Articles
Seminar or workshop
Lecture
Other
29- What challenges, if any, do you think physicians face when diagnosing and managing a concussion?
(An open-ended optional question)
